# Illuminating Human Norovirus: A Perspective on Disinfection of Water and Surfaces Using UVC, Norovirus Model Organisms, and Radiation Safety Considerations

**DOI:** 10.3390/pathogens11020226

**Published:** 2022-02-08

**Authors:** Richard M. Mariita, James H. Davis, Rajul V. Randive

**Affiliations:** Crystal IS Inc., an Asahi Kasei Company, 70 Cohoes Avenue, Green Island, NY 12183, USA; James.Davis@cisuvc.com (J.H.D.); Randive@cisuvc.com (R.V.R.)

**Keywords:** disinfection, LEDs, model organisms, human norovirus, physicochemical parameters, public health, radiation safety, UVC

## Abstract

Human noroviruses (HuNoVs) are a major cause of gastroenteritis and are associated with high morbidity because of their ability to survive in the environment and small inoculum size required for infection. Norovirus is transmitted through water, food, high touch-surfaces, and human-to-human contact. Ultraviolet Subtype C (UVC) light-emitting diodes (LEDs) can disrupt the norovirus transmission chain for water, food, and surfaces. Here, we illuminate considerations to be adhered to when picking norovirus surrogates for disinfection studies and shine light on effective use of UVC for norovirus infection control in water and air and validation for such systems and explore the blind spot of radiation safety considerations when using UVC disinfection strategies. This perspective also discusses the promise of UVC for norovirus mitigation to save and ease life.

## 1. Background

**Figure 1:**
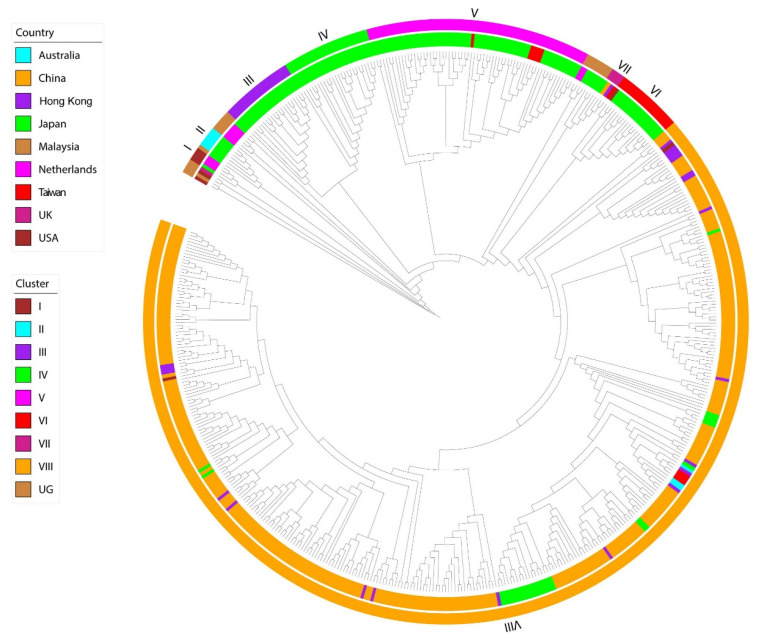
Phylogenetic diversity of HuNoV GII.2 strains based on major capsid protein VP1 depicting lineages in genogroups. Inner circle shows geographic location, while outer cycle shows phylogenetic clusters. Maximum likelihood was used to infer phylogeny using 519 *VP1* gene sequences. List of GenBank accession numbers have been provided in supplementary Table S1 of Li et al. [5]. Figure reused from Li et al. [5] under Creative Commons CC BY license http://creativecommons.org/publicdomain/zero/1.0/ (accessed on 11 January 2022).

Noroviruses (NoVs), belonging to family *Caliciviridae,* are positive-sense, single-stranded RNA viruses [1]. They are commonly spread through contaminated water and food and are a major cause of diarrheal illness [1,2]. The number of norovirus genogroups has been expanded to 10 (GI through GX) [3], five of which (GI, GII, GIV, GVIII, and GIX) comprise of human noroviruses (HuNoVs) [3]. Each genogroup is further subdivided into genotypes. GII.2 strains (Figure 1) have been reported as dominant isolates in recent outbreaks [4,5,6,7,8,9].

Annually, the global economic burden associated with gastroenteritis due to HuNoV is estimated to be more than USD 60 billion [10]. In the USA, norovirus gastroenteritis caused by HuNoV accounts for an estimated 56,000–71,000 hospitalizations and an average of 570–800 deaths per year [11]. To put it into perspective, in the USA, the HuNoV is associated with 20 times more gastroenteritis cases than any enteric pathogen [12]. Sometimes, the effects of norovirus are overlooked because they are often self-limiting in healthy adults, lasting a couple of days [13]. However, the effects of norovirus can be quite serious in young children, with over a million health care visits annually [14]. Additionally, for the elderly, HuNoV is associated with 20% of gastrointestinal (GI) deaths for those more than 65 years of age [15] as well as the immunocompromised, whose HuNoV infections can last for years [13]. Due to a wide diversity of norovirus genotypes [16], mutations, and the lack of good indicators of immunity, vaccine design, and development has been very difficult [2,17,18]. Therefore, prevention remains the best protection [19]. Still, the genetic, biological, antigenic, and antigen binding diversity, virulence, and stability in the environment of the HuNoVs make prevention difficult even for the developed countries [20,21]. Additional disinfection strategies, such as the use of UVC, will go a long way towards reducing infections and mortalities due to HuNoV.

## 2. Transmission of HuNoVs

Norovirus [22] is transmitted via surfaces [23], food contaminated by food handlers [24], contaminated vegetables, and undercooked or raw food [25], or water [26]. Person-to-person transmission is also unavoidable once a foodborne infection happens [25]. Transmissivity is high because it only requires a small inoculum size [27,28]. HuNoVs survive in the environment for a long time [27]. Disinfection has been critical to limiting transmission through the application of chlorine or other disinfectants in water [29], hand washing [23], and surface sterilization [23]. However, although norovirus can be transmitted year-round, hospitalizations and deaths peak in late winter and early spring [30]. It mainly spreads through fecal–oral transmission [31], either through person-to-person transmission or contaminated food or water [31]. It can also be transmitted through fomites [31]. Facilities that process food can greatly benefit by incorporating UV light technologies to control microbial transmission and ensure food safety [32,33].

## 3. HuNoV Control Programs

Norovirus outbreak management and disease prevention strategies are key in the disruption of disease transmission in many setting [27]. This can be in the form of institutional policies and development of effective hygiene programs and disinfection strategies, such as the use of UVC. In healthcare settings, guidelines aimed at preventing and controlling norovirus gastroenteritis outbreaks have been recommended [34]. These recommendations rely on empirical evidence although such evidence is limited by the inability to culture HuNoVs in laboratories [35]. Previously, there have been a few successful norovirus infection control tests in hospital settings [36,37,38]. For environmental health and safety, the current recommendation is physical removal of loose viral particles followed by use of medical grade disinfectants, especially ethanol, which has been used against both feline calicivirus (FCV) and murine norovirus (MNV). However, ethanol has limited efficacy against noroviruses [39], whereas bleach, although more effective at concentrations greater than 500 ppm [40], is limited to some surfaces, as it can cause corrosion of metals, craze plastics, and remove color from fabrics [41,42]. The CDC opinion on UV irradiation in environmental disinfection is that it remains an unresolved question [43].

There are also control programs meant to address food services [32,44] because they are such a frequent source of outbreaks. These programs focus on training managers and workers to identify warning signs, rigorously clean surfaces, wear gloves, and stay home when sick, all of which are useful protective practices [45]. Similar control programs already exist in some places for assisted living facilities and childcare facilities [45]. They generally involve very similar procedures—wipe down and clean surfaces, inform officials of any outbreaks, wash your hands, and wear gloves when cleaning vomit or feces.

The use of chemicals, such as bleach and ethanol, have limitations. For instance, occupational exposure to chemical disinfectants can cause lung damage [46]. Additionally, chemicals, such as hydrogen peroxide, peracetic acid, formaldehyde, sodium hypochlorite (household bleach), and beta-propiolactone, can be corrosive [47,48]. Further, observations have revealed that the disinfection performance of chemicals, such as chlorine and sodium hypochlorite, against norovirus surrogates is reduced in the presence of feces or other organic matter [49,50].

Minimal use of these chemicals, with the additional and safe use of UVC, will help curb HuNoVs. Still, for us to understand the disinfection efficacy of UVC, scientific evidence is needed. Unfortunately, at this time, model viral organisms (surrogates) are used to investigate disinfection efficacy, as HuNoVs, although cultivable [3], are challenging to use in this application (inactivation studies).

## 4. Surrogates: Considerations for Picking Norovirus Model Organisms for UVC Disinfection Studies

Previously, HuNoVs have been especially difficult to study because they could not be grown in tissue cultures [51]. Previous studies have involved acquiring samples from infected people, attempting to disinfect it with varying doses of disinfectants, and then intentionally infecting people through oral ingestion [52]. However, these studies are rare and cannot be used as the standard for measuring UV resistance or system design. Recently, developments on a tissue culture system for HuNoVs have been reported and used [53]. However, there are limitations to its use in inactivation studies, and the application is currently limited to human host-pathogen studies.

Thus, surrogates are almost always required for disinfection studies. Each has pros and cons, elaborated on in Table 1, which will be discussed further. Estimation of norovirus sensitivity to UVC radiation is from correlating UVC exposure’s impact on transcription using RT-qPCR or qPCR and cell cultures viability. RT-qPCR or qPCR results from surrogates’ studies, such as MNV, are then assumed to be similar in relationship to HuNoVs [12,54]. Given the circumstances, these are useful measurements. However, caution should be exercised during evaluation when using this approach, as RT-qPCR overestimates the number of infectious viral particles [55]. The viral reduction based on RT-qPCR will be lower due the overestimation of disinfected treatments.

It is also difficulty to account for differences between viruses or the impact of peak emission wavelengths of a UVC system.

Not all surrogates are equal. Thus, when picking the right surrogate for validation, environmental attributes, and genetic relatedness of surrogate to HuNoVs should not be the only attributes considered but other biological attributes as well [56,57]. For instance, HuNoVs can bind to histo-blood group antigens (HBGAs) in addition to infection and disease severity being associated with histo-blood group type [58,59]. Previous investigation has revealed that persons with blood group O phenotype are more likely to be infected with HuNoV, whereas those with B histo-blood group antigen have reduced risk of infection and disease symptom progression [58]. Surrogates, such as TV, bind to HBGAs [60]. Additionally, noroviruses bind to ligands beyond the histo-blood group antigens [61]. This includes receptors/co-factors (e.g., sialic acid), which can interact with the capsids of human norovirus [61,62]. Surrogates, such as FCV and MNV, bind to sialic acid on host cell surface [63,64]. A detailed review on of the two commonly used surrogates (FCV and MNV) from a food-safety perspective was published by Richards [65].

Although feline calicivirus (FCV) has been used as norovirus surrogate because of its cultivability, FCV is not an ideal candidate under some circumstances (Table 1). FCV is sensitive to low pH [72] and surface stress, including drying [85]. Therefore, if dried for UVC disinfection studies on dry surfaces, such as stainless steel, investigators need to consider the natural reduction due to the drying process. FCV also has different disinfection susceptibility under chlorine exposure [72,85] in addition to being more sensitive to UVC irradiation than HuNoVs [12].

On the other hand, murine norovirus (MNV) is an equally common surrogate for HuNoVs [69]. MNV is stable at a wider range of pH levels, is less sensitive to temperature, and shares genetic and molecular features with HuNoVs, such as size (diameter of 28–35 nm), icosahedral shape, and buoyant density (1.36 ± 0.04 g/cm^3^) [86,87,88]. However, MNV’s sensitivity to drying is like that of FCV, something that needs to be accounted for during validation. MNV is less sensitive to UVC than FCV and genetically closer to HuNoVs based on PCR studies [12,87].

Tulane virus (TV), the prototype recovirus strain, has been considered as a HuNoV surrogate [89]. Recovirus (ReCV) genus belongs to the family *Caliciviridae* and, just like HuNoV, is organized into three open reading frames (ORFs) [90,91]. TV recognizes the same ligand as HuNoV in oyster tissues [77,92]. The biological diversity and features of recovirus and clinical manifestations and disease progression in infected mammal organisms reflect HuNoVs properties more than with any prior surrogate [21]. Echovirus is like MNV in many ways and has been used as a surrogate [79]. MS2 has also been considered because it is a phage and easier to cultivate and test with. MS2 seems to be much more resistant than HuNoVs and so provides a large safety factor [12]. However, selecting a virus because of its high resilience may waste money and resources that could have been used protecting other pathways. Ultimately, when testing or designing new strategies for disinfection, one must understand and clearly justify the surrogate pick.

## 5. UVC Disinfection Studies That Utilized Norovirus Surrogates

Using deliberately contaminated stainless-steel surfaces, a previous study utilizing UVC at 260 nm to study the effects of 10–300 mWs/cm^2^ revealed that the murine norovirus-1 (MNV-1) titer was more sensitive than hepatitis A virus (HAV) [93]. The study also suggested that low doses of UVC can be used to decontaminated surfaces contaminated with HuNoV [93]. The effectiveness of UVC against MNV-1 has been associated with the disruption of the capsid protein and genomic RNA [75]. At 254 nm, with 25 mJ/cm^2^ UVC dose, a 3.6-log10 reduction was reported in a study carried out by Lee et al. [94]. This study also revealed higher MNV inactivation at high salt concentrations and temperatures (18 and 30 °C) compared to lower temperatures (−20 and 4 °C) [94].

Another study by Vimont et al. investigated the use of a pulsed-light device that emitted a broadband spectrum (200–1000 nm) and obtained a 3-log10 reduction using 3.45 J cm^−2^ in clear suspensions [95].

## 6. Considerations for Effective Use of UVC in Surface Disinfection

Per recent review by Raeiszadeh et al. [96], the COVID-19 pandemic has contributed to the increased consideration of UVC disinfection devices for disinfection. Proper use of UVC technology is required to guarantee disinfection when using devices such as Klaran WD array systems [97,98]. When disinfecting surfaces, considerations, such as type of surface (microstructural properties) being illuminated [99], intensity distribution projected onto target surface to be disinfected, and the distance between light source and target surfaces, should be factored into design and actual application. For instance, UVC disinfection may be more effective on plastic surfaces followed by stainless steel but lower on fabrics, such as poly-cotton [99].

One critical path for norovirus is through the food-service industry, where sick workers, contaminated water, or surface contact can contaminate food [25]. One strategy is to target all the pathways in the food-service industry. Since UV light can be used to disinfect surfaces and potentially even food surfaces, there is increasing interest for its use as a point-of-use disinfection method [32]. Examples of areas that could benefit from implementation of UVC technology include but are not limited to restrooms, kitchens, baggage claim areas, security checkpoints, and meeting rooms.

As discussed, surface transmission is one important pathway for norovirus outbreaks. However, before any research can be reasonably carried out, first, the methodology used during the study should be reviewed. The correct unit of light to be used to model UVC disinfection is fluence [100], or the amount of light through a sphere, divided by the cross section of said sphere. Studies that only measure exposure time and distance of UVC radiation from the surface being disinfected to determine disinfection efficacy of a system make universal application and replication hard and thus are insufficient. Although both time and distance are critical, they do not account for size, shape, reflection, temperature, or type of lamp, and therefore, results cannot be accurately transitioned into products/designs for real-world applications. Instead, fluence should be reported. For surfaces that absorb UV light, this simplifies from dose (mJ/cm^2^) to intensity (mW/cm^2^) multiplied by time (seconds) [101]. To ensure the correct measurement of the irradiance in experiments, it is best to use an optometer, such as the X1 MD-37-SC1–4, that is calibrated for the specific wavelength used in the experiment. Most optometers are designed for low-pressure mercury lamps, which radiate at 254 nm. Some of these, for example, will have a cut-off wavelength above 270 nm and are thus useless for measuring most LEDs or 222-nm filtered krypton lamps. Alternatives to optometers include potassium iodide acitometry [102,103], pre-existing disinfection systems with known irradiance at a well-defined point, and dosimeters selected for the correct wavelength [104].

In addition to the effect of the amount of light, the wavelength of light has a remarkable effect on disinfection rate. UVC LEDs have a peak wavelength between 215–280 nm, and that peak value can have a significant germicidal impact against many pathogens [105]. For example, with the same dose, 265–268 nm can increase disinfection of pathogens, such as SARS-CoV-2, by almost 1 log_10_ reduction value (1 LRV) compared to those with wavelengths >270 nm [106]. More studies are emphasizing that “not all wavelengths are created equal: [107,108]. Thus, measuring and specifying the wavelength and measurement tolerances used in a UVC system for disinfection is of critical importance for research, design, and implementation purposes. Spectrometers are available to perform such measurements, but spectrometers must be calibrated to ensure accurate intensities are reported.

The type of surface is also important during disinfection, as it has an impact on disinfection efficacy. The presence of small crevices can provide places for viruses to hide and avoid UVC exposure. On the other hand, reflecting surfaces can create numerous light passages, thus enhancing the disinfection efficiency [109]. For example, pathogens, including norovirus, on UVC-reflective materials, such as polished aluminum, may see 70% more light than measured with an optometer. That is because much of the light passes through the organism, but most will not be absorbed; it is then reflected through again. Since pathogens on porous absorbing materials may be shielded from most UVC light, lower inactivation efficacies are obtained. In surface disinfection, such reflection or coverage is accounted for simply by testing on different materials.

Physicochemical properties also impact UVC disinfection. Often, relative humidity is considered and reported only for studies involving aerosolized microorganisms. However, relative humidity effects the kill rate of viruses on surfaces and not just in air [110,111]. Temperature and humidity can affect survival of viruses [109].

Another limitation is around the way we consider populations. Often, we discuss log reduction values as simple additive reductions. This assumes that all viruses are the same and that if an exposure was able to kill, for instance, 90% in the first 10 s, then a 90% reduction within same time means identical results. This is erroneous because even if all the viruses were of the same exact genome, damage, if any, and micro-environment, which is far from certain, some viruses may be shaded, and others may interact with surfaces differently. For instance, 99.9% reduction within 10 s may be a reasonable approximation; it can be broken down at both low and high doses of any disinfectant, including UVC [112]. Therefore, for applications with either high, low, or a high dynamic range of dose, a log-normal distribution may be needed.

The ideal test of a norovirus surface disinfection system or study of sensitivity would cover a variety of relevant surface materials, with many different environmental conditions, with known, widely varied wavelength and fluence against the right surrogate. However, to the best of the authors’ knowledge, there is no such complete study for a norovirus because of the time and resources that are required to conduct such a study.

Autonomous navigation robots capable of recognizing surfaces with high probabilities of contamination are emerging [113]. It will be tremendous if such robots in future can detect the microstructural differences and adjust UVC dose accordingly. While these autonomous navigation robots are at work, it will be important that they have a feature to determine safe distance between them and nearest human beings and be able to warn them to be out of the way while disinfection is ongoing. They should be equipped with safety features, such as motion sensor, and be able to turn off UVC radiation when humans or animals approach [114] and wait for humans to move before proceeding with their disinfection work. Companies that make these products should ensure adequate safety features to protect humans against exposure to unsafe amount of UVC, as that could lead to advisories from guidance, compliance, and regulatory bodies in addition to other consequences [115,116].

## 7. Considerations for Conducting Water Disinfection Using UVC

Water disinfection has a great deal in common with surface disinfection. Fluence and wavelength are both still important, as are environmental factors. However, the number and types of environmental factors are different. Water disinfection reactors (flow-through systems with water exposed to UVC radiation) and water tanks do not always have reflective material and are almost always sealed. Water disinfection reactors mostly use reflective materials, such as polytetrafluoroethylene (PTFE), to enhance disinfection. During experiments, it is hard to measure dose (mJ/cm^2^) straightaway. To that end, inserting an optometer through a small hole or partially disassembling a system may be required. If so, then the hole should be as small as possible and correctly account for reflection multiply by 1 + R [117,118], with R being reflectivity of the inner reactor material. Another alternative is to use potassium iodide actinometry, but one must account for the UVC absorption [101].

Turbidity and absorption of fluid due to high concentration of Total Dissolved Solids (TDS), for instance, is one such different aspect that can impact inactivation efficacy [119]. While transmitting through air onto a surface, air is generally assumed to be 100% transparent, with low turbidity. None of the light passing through the air will be deflected away from the virus or be absorbed by gas. On the other hand, in liquid, it is possible to be turbid and absorbing to the extent of making a system completely ineffective, as has been demonstrated in aquacultures [120]. Hence, measurement of the UVC transmission and turbidity are needed. Noroviruses are also sensitive to pH [49,73]. Therefore, ethanol-containing acidic alcohol suspensions (e.g., 70% ethanol containing 1% citric acid) have more virucidal effect compared to 70% ethanol [121]. pH should always be reported as a factor during studies where liquids are involved. Just like on surfaces, norovirus deactivation rates also depend on temperature. It is vital to measure and control the temperature and pH of water during disinfection tests, as they directly influence the dynamics and distribution of other microbial assemblages [57,94,122].

Additionally, water supplies can have varying concentrations of chlorine or other disinfectant residuals. Some of these disinfectants are typically activated by UVC for various reasons but often through the formation of chlorine or oxygen radicals [123]. Therefore, it is critical to know the concentration of disinfectants in a test and recommended to design the study around them.

Furthermore, there is virus protection against UVC inactivation that could be expected when viral particles are in fecal matter or environment with organic matrix [124], which could lead to low viral inactivation efficacies by UVC. This has been observed when other agents are used in norovirus disinfection [125]. It is therefore important to consider properties of the materials to be disinfected. For the users to consider using UVC for norovirus infection control, they need to be guaranteed that the expected performances are obtained. Thus, this calls for a focus on the principles of disinfection and decontamination during UVC implementation toward norovirus infection control [35]. It is also important to consider if the type of water to be disinfected is static or flowing, as different types of water behave differently. All water is not equal; thus, the state (static or mobile) and physical as well as chemical composition of water will affect microbial stability and UVC dose requirements [126].

## 8. Radiation Safety Considerations

UVC light has safety concerns that prevent certain uses or require safety considerations during use [96]. Firstly, as a fact, UVC and even far UVC does not discriminate between pathogens and commensals. Because of that non-discriminatory nature and potential impact on the skin’s microbiome, any UV could induce human immune suppression [127]. It is important to know that commensals, especially those of the human skin surface, are crucial, as they induce protective responses that defend us against invasion and colonization by pathogens. The role of commensals (symbionts) in protective immunity has reviewed by Khan et al. [128], Li et al. [129], Byrd et al. [130], Grice and Segre [131], and Flowers and Segre [132].

The human skin microbiome has adapted to consume sparse nutrients available on our skin, in exchange for protecting us [130]. Although known to be stable regardless of perturbations, the kind of imbalance due to inactivation by UVC has not been studied. We predict that that due to dysbiosis (reduction of microbial diversity of beneficial bacteria due to indiscriminate inactivation), selection pressure, and enrichment of bacteria with high guanine–cytosine (%GC) composition bacteria on skin, we could potentially see an explosion of skin diseases and related conditions if exposure is for long period of time. Bacteria with high %GC, such as Actinobacteria, are more tolerant to UV [133]. If not safely implemented, UVC exposure at the airports when moving from gate A to Z or while waiting for hours to board the aircraft could enrich for high %GC content bacteria while inactivating beneficial ones with low %GC. Another example is in schools or hospitals, where there will be more exposure time. It will therefore be important that facility managers understand the spaces where the UVC can be safely and effectively implemented.

Since UVC is indiscriminate, it can damage all living cells it reaches. These cells are not restricted to mammalian cells but also bacteria and viruses for that matter. Although the good viruses that form part of the skin microbiome are poorly understood, they act as primary barrier to the external environment and help humans modulate cutaneous health [134]. We emphasize this because of the arguments that 222 nm is safe without regard for the critical role of the skin microbiome and potential for directed evolution of UVC resistant pathogens. Although erythema can be absent, irradiation at 500 mJ/cm^2^ using 222 nm UVC does decrease the number of bacteria while producing cyclobutene pyrimidine dimers (CPD) [135].

In fact, skin exposure can cause cancer with the right dose at almost all UVC wavelengths [136]. It can also cause retinal cancer and blindness [137]. It is reasonable that there is increasing interest in 222 nm, as it may not reach through the endoplasm of large cells and even less through dead skin cells or the lens of the eye, thus reducing the risk of cancer and blindness. As a result, 222-nm lamps are increasingly being used in open environments [43,138]. Although the promise of disinfecting an entire restaurant continuously to reduce norovirus spread sounds promising, several issues raised in this perspective should be considered during applications.

The other important concern is that although some studies show little to no corneal or skin damage at exposures of 100 s mJ/cm^2^ [138] from 222-nm light, they still show damage at doses of 1000 s mJ/cm^2^ [138]. Although there is hope that this effect is not cumulative, as epithelial shedding may provide added protection, this has not yet been tested, to the best of our knowledge. There has also been very little testing on humans—although one author exposed their eye briefly and informally and reported only mild irritation [139]. There has also been little consideration for variations like dry eyes, which may remove a critical protective barrier; cuts or abrasion of the skin, which may remove protective dead skin cells; or exposure to other sensitive areas. Thus, it is concerning to suggest that far UVC should be applied in airports, hospitals, schools, and homes with constant or long exposure time [43,138].

For emphasis, a dose of 100 mJ/cm^2^ at 222 nm reduces all pathogens by several orders of magnitude, hopefully without harming human cells [44]. However, just like with antibiotics, it should be noted from a skin microbiome perspective that the use of 222 nm or any far UVC on skin in general will not discriminate between pathogens and the “good” skin microbiome that is required for healthy skin. Skin microorganisms have important roles in the cutaneous immune system [130]. These “good” microbes also produce molecules that prevent skin colonization from other unwanted microorganisms or protect us by altering the behavior of the invaders, ensuring the protection and health of our biggest organ, the skin [130].

We therefore expect that indiscriminate frequent exposure of human skin to 222-nm light will likely inactivate symbionts and unbalance the taxonomic, phylogenetic, and functional potential of the microbiome, leading to a compromised cutaneous immune system, the consequence of which will be skin disorders, such as acne. The enrichment of UVC resistant is most likely to be for those skin microbes like *Propionibacterium acnes,* whose %GC content is high [45], and have less likelihood of forming enough dimers for inactivation. UVC application on skin will likely reduce microbial diversity due to inactivation of high AT commensals. A reduction of microbial diversity due to cutaneous microbial dysbiosis could lead to other conditions, such as psoriasis [140].

The demodex mites are part of normal skin flora and feed on microbes, such as *P. acnes* and sebum [131]. We speculate a taxonomic imbalance, which will in turn lead to functional imbalance, impacting immunity, and perhaps skin syndromes. Molecular, immunological metabolomic, and microbiological investigations are recommended in the future to unearth any potential functional dysregulation due to UVC usage on skin.

Regardless of the wavelength, the process for safe work with UVC light is similar. Wear long sleeves and pants that cover your whole body. Wear plastic or close-stitch gloves to absorb UVC light, which is also helpful in preventing smudges on your light source or reflectors. Wear plastic goggles to absorb UVC light. Most light sources will be sensitive to electro-static discharge (ESD), so consider wearing ESD shoes, bracelets, or boot covers. Some light sources require high voltages—such sources should not be used near flammables and should be isolated from users before powering on. Some light sources are made with glass and contain hazardous chemicals, so after use or breakage, follow the manufacturer’s disposal instructions.

The design and implementation of UVC disinfection reactors or sources should strive to prevent stray light from escaping. This can be done in water reactors by placing them ahead of a tubing of a material that will absorb UVC, such as most plastics and glasses. This forms a simple light baffle, which will absorb light but merely direct the water. A more complex tube shape, such as a u-bend or a mesh, can shorten the required distance. On surfaces, it is best if any design physically contacting the surface to be disinfected contains the light there.

The concerning potential disadvantage of the UVC technology involves potential dysregulation of both taxonomic, phylogenetic, and functional diversity of the skin microbiome. A survey on the composition, structure, and functional changes is thus recommended. For UVC to be effective, the target pathogens must be in direct contact with the UVC irradiation. Fortunately, UVC dosimeters, if correctly used, can be used for verification of UVC exposure [141]. Moreover, UVC can cause harmful effects on eyes and skin, and it is therefore not safe for use in disinfecting hands and skin [115,142].

## 9. The Promise of UVC for Norovirus

UVC disinfection of surfaces could have several critical norovirus-related applications. For example, washing fruit and vegetables may accidentally inoculate norovirus to surfaces. Additionally, chlorine is not allowed in many European countries and rarely desired anywhere because of its effect on the food and its residue both in the food and waste. However, recent studies have shown that food processing, which combines washing and application of UVC, can enable lighting all the relevant surfaces of various fresh fruits and vegetables [32,75,143,144,145,146]. UVC disinfection also promises an explosion hazard-free and chlorine-free way of disinfecting counter-tops, preparation areas, bathrooms, and any other high-touch surfaces repeatedly with little to no labor.

UVC could be and increasingly is used to disinfect wastewater to provide a low-impact way of protecting water estuaries and seafood [147,148,149]. Like with surface disinfection, there is significant interest in the synergy between TiO_2_ [147], H_2_O_2_, and Cl_2_ [150] with UVC to further improve disinfection performance. Such improvements may reduce the amount of time, power, and disinfectant required.

However, a thorough measurement of UVC disinfection efficiency of norovirus and its primary surrogate MNV in different environmental conditions would make design and implementation of UVC disinfection systems easier for water and surface disinfection. Exploring the factors of temperature, pH, humidity, wavelength, and surface type would allow designers to account for various situations in implementing their disinfection systems. Larger-scale adoption of UVC may still depend on both low barrier to entry and greater visibility of the benefits of disinfection.

UVC disinfection research, although eliminating some pathways, does not address many other problems. Many outbreaks stem from worker cleanliness and delayed detection. However, UVC could also play a role in tackling this difficult problem through timed auto-fluorescent measurement of pathogens [151,152,153]. Such systems might provide a method of scaling up point of outbreak monitoring by allowing, for example, restaurants, schools, and long-term care facilities to check for infection every shift. This could dramatically reduce the number of people exposed and the amount of exposure in an outbreak. However, such systems require significant further development—both in tools, such as high-sensitivity UVC detectors, high-speed switching UVC LEDs at multiple bands, analyzers, and databases, and more.

The 222 nm is not yet proven for long-term exposure in any surrogates and has not been tested in humans. However, such research, if proven safe, may provide a new route to continuous disinfection. Still, even if use of UVC does not prove safe in specific cases, it may still be useful in quickly dosing unoccupied public areas—especially in conjunction with other disinfectants, which may allow for more effective cleaning. Although traditional UVC sources, such as mercury lamps emitting UVC radiation at a peak wavelength at 254 nm, can be used, it is important to note that the overall log-inactivation efficacy at peak emission wavelength at 265 nm has been found to be high and most effective [154,155]. Furthermore, the use of low-pressure (LP) mercury lamps emitting radiation at 254 nm poses environmental concerns due to mercury content [156].

## 10. Conclusions

We have a long way to go in the fight against human norovirus. Perhaps the most important and exciting research direction is in developing methods for culture, growth, and measurement of HuNoVs because these developments underpin all others. However, the development of convenient detection may also reduce the outbreak time, while UVC disinfection of foods and services will help curb infectious pathways.

Until proven and the impact on the skin microbiome understood, shining UVC directly into occupied space should be avoided, as UVC does not have a mechanism to discriminate between pathogens and commensals, which are critical to our own health. Surveys utilizing shotgun metagenomics could help in evaluating the impact on commensals and associated changes in taxonomic and functional dynamics, including changes in secondary metabolites’ production.

## Figures and Tables

**Table 1:** Comparison of some human norovirus (HuNoV) model organisms (surrogates).

Surrogate	Advantages	Disadvantages	Source
Feline calicivirus (FCV)	− Cultivable − Stable at some temperatures − Frequently used as surrogate in chemical disinfection and irradiation studies − Like HuNoVs, found to remain infective beyond 70 days on stainless steel and plastic at room temperature	− RT-PCR results indicate FCV is easier to inactivate (e.g compared to MNV and MS2) and should be used as surrogate with caution − Sensitive to low pH unlike HuNoVs − Sensitive to drying − Different transmission route (FCV is a respiratory and not an enteric virus link HuNoV) − High infectivity-reduction rate	[49,65,66,67,68,69,70,71,72,73,74]
Murine norovirus (MN)	− Cultivable − Stable across the a wide pH range. − Genetic similarity and environmental stability comparable to HuNoVs − Commonly used as surrogate in chemical disinfection and irradiation studies − Like HuNoVs, found to remain infective beyond 70 days on stainless steel and plastic at room temperature − Conservative HuNoV surrogate for UV disinfection	− Sensitive to drying − Different UVC susceptibility to other surrogates − MNV environmental stability is different to HuNoV	[57,65,66,68,69,70,75,76]
Tulane virus (TV)	− Cultivable − The biological features of recovirus closely reflect HuNoVs − Clinical manifestations and disease progression in infected mammalian organisms reflect human norovirus − Truly intestinal pathogen, also from *Caliciviridae* family like HuNoV − Recognizes the same ligand as HuNoVs − More resistant at low pH than FCV	− Difficult to produce a high-titer stock via tissue culture − Reduction of virus titer at pH 2.5 and 9.0	[21,69,77,78]
Echovirus 12	− Cultivable − Conservative HuNoV surrogate for UV disinfection − Shares morphological similarities with HuNoVs − Environmental persistence − Poses lower human health risks	− This is a BSL-2 organism and may not be used widely. − Long incubation period (2–5 days)	[79,80,81]
MS2 and Qβ	− Cultivable to high titers − BSL-1 organisms (non-pathogenic), thus possibility of widespread use − Potential surrogate for noroviruses on fresh produce with prolonged survival periods, both in buffer and on fresh produce, at temperatures relevant to chilled foods. − Conservative disinfection performance due to high UVC sensitivity than most pathogenic viruses	− Sensitive in acidic environments − UVC resistance may lead to resource wasting, as it is too conservative.	[71,82,83,84]

## Data Availability

Data sharing not applicable. No new data were created or analyzed in this study.
